# Examining the relationship between anthropometric and body composition measures with coronary heart disease events: the multi-ethnic study of atherosclerosis

**DOI:** 10.1093/eurjpc/zwaf212

**Published:** 2026-10-13

**Authors:** Amier Haidar, Richard Kronmal, Matthew Allison, Karol Watson, Preethi Srikanthan, Tamara Horwich

**Affiliations:** 1 Division of Cardiology, David Geffen School of Medicine at UCLA, 10833 LeConte Ave CHS A2- 237, Los Angeles, CA 90095, USA; 2 Department of Biostatistics, University of Washington, Seattle, WA, USA; 3 Department of Medicine, University of California at San Diego, San Diego, CA, USA

**Keywords:** Fat mass, Fat-free mass, Waist circumference, Waist-to-height ratio, Coronary heart disease

## Abstract

**Aims:**

Obesity, traditionally measured using body mass index (BMI), is considered a significant risk factor for the development of cardiovascular disease (CVD). However, there have been mixed findings regarding the association between obesity and cardiovascular events in part due to the way it is measured. Major limitations exist with the use of BMI as it cannot assess body composition or fat distribution, and thus fails to account for the separate roles of muscle and fat mass (FM). The objective is to examine the relationship between various measures of body composition including FM, fat-free mass (FFM), waist-to-height ratio, waist circumference, and BMI, and coronary heart disease (CHD) events, from the Multi-Ethnic Study of Atherosclerosis (MESA) cohort.

**Methods and results:**

MESA is a population-based cohort study of 6814 men and women, aged between 45 and 85 years, without clinically apparent CVD. FM and FFM at baseline (Exam 1) were estimated using multiplicative power equations formulated from bioelectrical impedance analysis (BIA) measurements. Multi-variable Cox regression models were used to estimate the hazard ratio (HR) and 95% confidence interval (CI) for 10-year incident hard CHD events (non-fatal myocardial infarction, resuscitated cardiac arrest, and death resulting from CHD) for FM/FFM, waist-to-height ratio, waist circumference, and BMI. Higher FM was associated with higher risk of hard CHD events, with a HR of 1.64 (1.09–2.48) per standard deviation (SD) unit increase (*P*-value 0.019). FFM was associated with a lower risk of CHD, with a HR of 0.24 (0.08–0.76) per SD unit increase of FFM (*P*-value 0.014). The waist-to-height ratio had a HR of 1.21 (1.08–1.35) (*P*-value 0.001) per SD unit increase in the ratio and was significantly associated with a higher risk of hard CHD events, as was waist circumference with a HR of 1.14 (1.03–1.28) (*P*-value 0.016) per SD unit increase. BMI had a HR of 1.08 (0.97–1.21) (*P*-value 0.158) and was not significantly associated with a 10-year risk of hard CHD events.

**Conclusion:**

Higher FM, waist-to-height ratio, and waist circumference were associated with increased risk for hard CHD events while higher FFM was associated with decreased risk. This highlights the potential importance of lifestyle interventions to build muscle and minimize adiposity for the prevention of CVD and strengthens the call for the inclusion of alternative methodologies to define obesity and measure body composition in the clinical setting.

## Lay Summary

Examining the relationship between various measures of body composition including estimated fat mass (FM) and fat-free mass (FFM), waist-to-height ratio, waist circumference, and BMI, and coronary heart disease (CHD) events.

- Higher FM, waist-to-height ratio, and waist circumference were each associated with increased risk for hard CHD events.
- Higher FFM was associated with decreased risk for hard CHD events.
- Combining both waist-to-height ratio and FFM measures in predictive models most accurately predicted future risk of CHD events

## Graphical Abstract

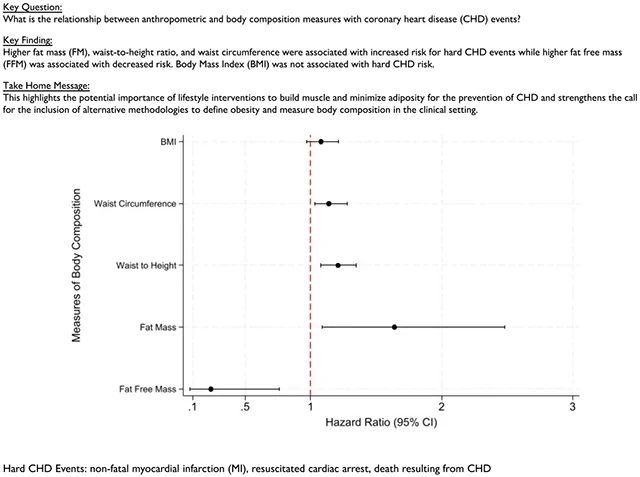

## Introduction

Obesity, a significant risk factor for the development of cardiovascular disease (CVD), is a major public health concern in the United States and has increased in prevalence over the past 20 years.^1-4^ Obesity is traditionally defined as excess body fat accumulation and is most commonly clinically measured using body mass index (BMI).^5,6^ However, there have been disparate results from assessments of CVD risk by BMI, such that while in some studies BMI has been strongly associated with an increased risk of CVD events, CVD mortality, and all-cause mortality, in other studies the association between CVD and BMI has been noted to be paradoxical or U shaped.^7,8^

The inconsistent association between BMI-defined obesity and CVD risk likely stems from the inability of BMI to characterize body composition, i.e. quantity, quality, and distribution of fat and lean mass, all of which are important components of cardiometabolic risk.^4,9-11^ For example, visceral and ectopic fat are highly associated with cardiometabolic risk while subcutaneous fat is not.^4,11-20^ Metrics of abdominal fat such as waist circumference and waist-to-height ratio have thus been proposed as useful measures to refine cardiometabolic risk in the definition of obesity.^21,22^

There have been mixed findings regarding the association between fat mass (FM) and cardiovascular events.^13,14,16,23-29^ Both body fat distribution and the phenotype of the adipose tissue play a critical role in determining cardiometabolic risk. Most studies show decreased muscle mass and increased adiposity, particularly truncal adiposity, to be associated with adverse cardiovascular events,^13,14,23,24,27,29-33^ while a few have either found either a different relationship or no relationship.^25,26^ Thus, the objective of the present study was to examine the relationship between measures of body composition including FM, fat-free mass (FFM), compared with relevant anthropometric measures including waist-to-height ratio, waist circumference, and BMI, with coronary heart disease (CHD) events, using participant data from the Multi-Ethnic Study of Atherosclerosis (MESA).

## Methods

### Study design and population

MESA is a population-based, prospective cohort study of 6814 men and women from the following 6 US communities: Baltimore City and Baltimore County, Maryland; Chicago, Illinois; Forsyth County, North Carolina; Los Angeles County, California; Northern Manhattan and the Bronx, New York; and St. Paul, Minnesota. Baseline data were collected from July 2000 and August 2002 (Exam 1): the participants were aged between 45 and 84 years and without clinically apparent CVD. The study protocol was approved by the institutional review boards at all field centres. During the baseline exam all participants provided written informed consent, and the participants were invited to complete subsequent exams and questionnaires. Further details of the study design have previously been described.^34^

### Measurement of covariates

Standardized questionnaires were used were used to ascertain age, sex, race and ethnicity, education and income levels, occupational information, smoking status, medical history, physical activity, and medication use for diabetes, lipid lowering, and hypertension. Height was measured with a stadiometer and body weight with a balance scale to the nearest 0.1 cm and 0.5 kg, respectively. Waist circumference was measured to the nearest 0.1 cm, at the minimum abdominal girth using a steel measuring tape with standard 4-ounce tension. Body mass index (BMI) was calculated by dividing weight in kilogram by height in meters squared. Seated blood pressure was measured three times at 1-min intervals following a standardized protocol. The average of the last two measurements was used for analysis. Total and high-density lipoprotein cholesterol, and triglycerides were measured at a central laboratory (University of Vermont, Burlington, Vermont).

### Coronary heart disease events

Events and mortality were verified with medical records or interviews by two physicians from MESA. Incident hard CHD events included non-fatal myocardial infarction (MI), resuscitated cardiac arrest, and death resulting from CHD. Incident CHD events were adjudicated by the MESA mortality and morbidity review committee. MI required either abnormal cardiac biomarkers (more than two times upper limits of normal) regardless of chest pain or ECG findings; evolving Q waves regardless of chest pain or biomarker findings; or a combination of chest pain, and ST-T evolution or new LBBB, and biomarker levels one to two times upper limits of normal. Resuscitated cardiac arrest was classified when a patient successfully recovered from a full cardiac arrest through cardiopulmonary resuscitation. Fatal CHD required a documented MI within the previous 28 days, chest pain within the 72 h before death, or a history of CHD, and required the absence of a known non-atherosclerotic or non-cardiac cause of death.

### Body composition

**Figure 1:**
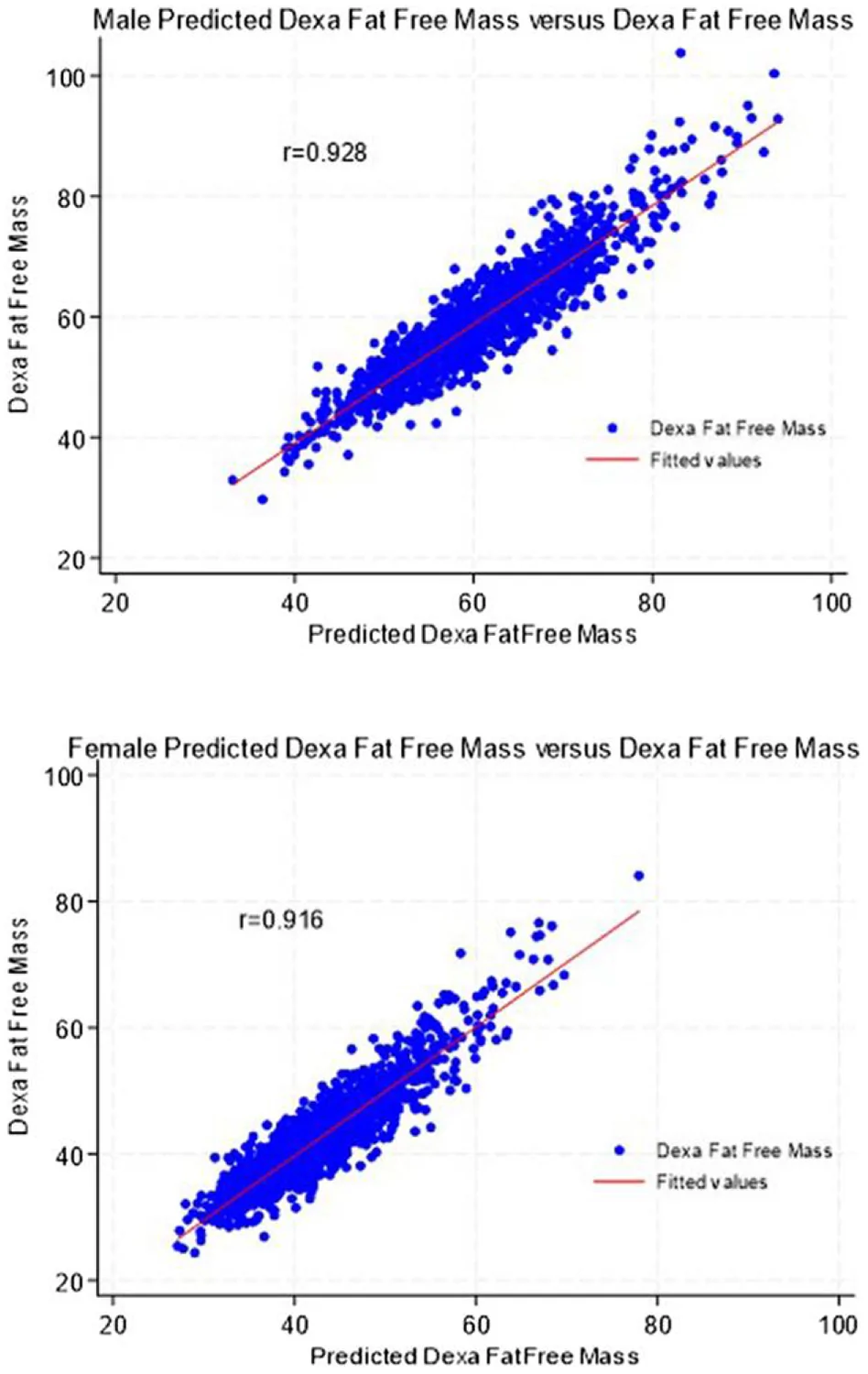
Plot of the estimated National Health and Nutrition Examination Survey (NHANES) fat-free mass (FFM) vs. the dual-energy x-ray absorptiometry (DEXA) NHANES FFM for males and females.

Multiplicative power equations were used to estimate FFM. In MESA Exam 5, participants bioelectrical impedance analysis (BIA) FFM was measured using a Valhalla BIA scale. Linear regression was used to predict the log of the BIA FFM with logs of the dependent variables: weight, height, and age [e.g. $\log(\text{BIA FFM})=\beta_{0}+\beta_{1}\log(\text{weight})+\beta_{2}\;\log(\text{height})+\beta_{3}\;\log(\text{age})]$]. Separate equations were estimated for males and females. Exponentiating the log equation provides the FFM estimating equation: $\text{FFM}=\text{exp}(B_{0})*{\text{wight}}^{B_{1}}*{\text{height}}^{B_{2}}*{\text{age}}^{B_{3}}$, where $B_{0}$, $B_{1}$, $B_{2}$, $B_{3}$ are the estimates of the $\beta^{\prime }s$. The estimation equations are: Male $\text{FFM}=6.690*({\text{age}}^{-0.180})*({\text{weight}}^{0.491}*({\text{height}}^{1.397})$ and female $\text{FFM}=8.668*({\text{age}}^{-0.248})*({\text{weight}}^{0.499}*({\text{height}}^{1.026})$. The FM is estimated as weight—FFM. To validate the FFM equations, the National Health and Nutrition Examination Survey (NHANES) 2003 dual-energy x-ray absorptiometry (DEXA) FFM values are compared with FFM estimated using the MESA equations and the weight, height and age for the NHANES 2003 participants. The correlation of the MESA estimated FFM with NHANES DEXA FFM was used as a measure of agreement. Plots of the MESA estimated FFM vs. the DEXA FFM are shown (Figure 1).

### Statistical analysis

Continuous variables were expressed as means and standard deviations, while frequencies and percentages were determined for categorical variables. The survival analyses evaluation period was from the MESA baseline exam to 10 years of follow-up.^35^ Multi-variable Cox hazard regression models were used to estimate the hazard ratio (HR) and 95% confidence interval (CI) for 10-year incident hard CHD events for FM/FFM, waist-to-height ratio, waist circumference, and BMI. Likelihood χ^2^ tests (LR χ^2^) were calculated to compare the models. Covariates included in the final model were those in the MESA risk score and included age, gender, race/ethnicity, smoking history, LDL cholesterol levels, HDL cholesterol levels, diabetes status, systolic blood pressure, anti-hypertension medications, and family history of heart attack.^35^ A model that included interactions of the age, gender, and ethnicity with both FFM and FM was run, and the coefficients were tested for statistical significance. An additional Cox model was run with measures of income, education, and exercise included. Restricted cubic splines were used to explore potential non-linear relationships between anthropometric and body composition measures and outcomes. Tests for non-linearity of FM, FFM, BMI, WC, and waist-to-height ratio were not statistically significant. In all analyses, *P*-values < 0.05 were considered statistically significant. STATA 18 was used for all analyses.

## Results

The means and CIs for age, weight, and height in MESA Exam 1, Mesa Exam 5, and NHANES 2003 participants are shown in Supplementary material online, Table S1. The estimated MESA baseline FFM and the DEXA FFM are shown in Table 1, providing evidence that the MESA and NHANES samples were similar. The correlations between the DXA estimated FFM in the NHANES 2003 population, compared with the FFM from the MESA derived equations, were 0.92 for both males and females, demonstrating an external validation of the MESA FFM equation (Figure 1).

Descriptive characteristics for MESA Exam 1 participants are displayed in Table 2. There were 3585 females and 3200 males. The average weight was 73.71 kg for females and 84.13 for males. Mean FM for females was 30.66 and 21.67 kg for males. The average waist-to-height ratio for females was 0.61 and 0.57 for males. The average age of the study sample was 62 years, and 38.5% were White, 11.8% Chinese American, 27.7% African American, and 22% Hispanic American.

Measures of body composition and their associations with hard CHD events are presented in Table 3 (standardized in SD units); see Supplementary material online, Table S2 for results in original units. Anthropometric measurements categorized in quartiles are presented in Supplementary material online, Table S3. Estimated FM and FFM were significantly associated with hard CHD events in the fully adjusted multi-variable Cox regression model. Higher estimated FM was associated with higher risk of hard CHD events, with a HR of 1.64 (1.09–2.48) per SD unit increase in FM (*P*-value 0.019). On the other hand, estimated FFM was associated with a lower risk of CHD, with a HR of 0.24 (0.08–0.76) per SD unit decrease in FFM (*P*-value 0.014). Diabetes, smoking history, LDL cholesterol, systolic blood pressure, and family history were also associated with CHD. There was no evidence of confounding for any of the clinical or behaviour variables when comparing the coefficients with the model that included FM and FFM to the MESA model without the body composition variables.

Waist-to-height ratio, with a HR of 1.21 (1.08–1.35) (*P*-value 0.001), was significantly associated with a higher risk of hard CHD events. BMI had a HR of 1.08 (0.97–1.21) (*P*-value 0.158) and was not significantly associated with a 10-year risk of hard CHD events. Waist circumference had a HR of 1.14 (1.03–1.28) (*P*-value 0.016) and was significantly associated with a 10-year risk of hard CHD events. The models that included FFM and waist circumference or waist-to-height ratio had the highest LR χ^2^ of 358.55 and 358.24, respectively.

## Discussion

The results from this prospective cohort analysis of MESA indicate that at baseline, waist-to-height ratio, waist circumference, estimated FM, and estimated FFM were significant predictors of hard CHD events. Patients with greater FM had higher 10-year risk of hard CHD events, while FFM was associated with lower 10-year risk of hard CHD events. Patients with a higher waist-to-height ratio and a greater waist circumference also had a higher 10-year risk of hard CHD events. Notably, BMI was not significantly associated with incident hard CHD risk. Additionally, in this study estimated FM and FFM were determined using generalized equations created from BIA data (MESA Exam 5) that demonstrated strong external validity when applied to the NHANES 2003 dataset.

The most widely used measure for adiposity for research and clinical purposes are BMI. While quick and easily assessable, it has many limitations as it cannot differentiate between fat and muscle mass quantity, quality, and distribution, and does not account for differences in body composition influenced by age, sex, weight, and height, and thus is a poor prognostic indicator of obesity when compared with waist-to-height ratio, WC, FFM, and FM.^5,6,36-38^ Given the limitations, BMI is a less ideal anthropometric index in the assessment of CVD risk as it cannot consider the individual role of both muscle and adiposity.^4,9,10,39^ Fat and FFM estimation, compared with the weight-based BMI, may allow for increased accuracy and improved risk stratification.^5,6^

Bioelectrical impedance analysis (BIA) is the most common method for determining FM and FFM.^40^ While BIA is becoming more commonplace in clinical practice, there is large variability between devices and a lack of standardization.^41-43^ Difficulties exist in the validation of BIA in different age and ethnic groups, and clinical conditions with abnormal hydration states, which has resulted in a plethora of BIA equations that may confuse, rather than aid in the interpretation of BIA results.^42^ Additionally, although many equations have been published for determining BIA, the variables of height, weight, age, and sex account for most of the variance in BIA estimated FFM and FM.^41,42,44^ Predictions of FFM based on models derived from physical characteristics have been shown to be similar to DEXA determinations.^41^ The use of equations in the current study derived from sex, age, weight, and height is significant to the study of body composition and obesity, as these equations were validated with NHANES DEXA data and shown to have generalizability across ethnic groups. The utility of the equations developed in this study provides a potential novel clinical and research tool for estimating body composition and cardiovascular risk without the need for a BIA device or DEXA scan.

In the current study, estimated FM was significantly associated with increased 10-year risk for the development of hard CHD events. This study adds to the literature by further elucidating this complex relationship.^14,16,23,24,26,28^ Multiple studies have shown a strong positive association between FM and risk of CHD or MACE.^14,16,23^ Medina-Inojosa *et al.* demonstrated that in patients with CAD, higher body fat percentage, as assessed by air displacement plethysmography, was associated with high risk of major adverse cardiovascular events (MACE).^23^ Another study, among UK biobank participants, also showed a strong positive association between BIA-derived FM and CVD risk.^16^ Similar to the present study, a study of Korean adults aged 40–79, who were free from stroke and CHD, found DEXA-derived FM to be significantly associated with higher 10-year CHD risk.^14^ Of note, a few studies have also shown that FM is not associated with CVD risk.^24,26,28^ Ohori *et al.* concluded that high percent body FM was associated with lower risk of short-term cardiac events in HF patients.^28^ In this study, it is noteworthy that 67% of enrolled patients exhibited muscle wasting, thus it can be speculated as to whether the level of FM or associated decline in FFM account for the positive relationship with cardiac events. Two studies demonstrated no relationship between FM and MACE in patients with CAD.^24,26^ All three studies that did not find a positive relationship between FM and CV events included participants with pre-existing CVD or HF, whereas MESA participants were free of clinically apparent CVD at baseline. Overall, these studies demonstrate that in patients without pre-existing CVD, FM, regardless of the method used to quantify it, is associated with increased 10-year risk for CVD events. Fat reduction, through whole food, Mediterranean-style diet, and physical activity, has been shown to improve metabolic health and reduce CVD risk.^45-47^

The current study also found that estimated FFM was significantly, negatively associated with 10-year risk for the development of hard CHD events. This finding is in accordance with the existing body of literature that has demonstrated an inverse relationship between FFM and CVD risk.^14,23,24,30-33^ In prior studies, FFM was determined using CT scans,^31,32^ BIA,^33^ DEXA,^14^ air displacement plethysmography,^23^ and validated equations.^24,30^ In all studies, FFM was significantly inversely associated with the risk of MACE. Since muscle is the primary tissue contributing to whole-body insulin-mediated glucose disposal, muscle has a metabolically protective effect, and individuals with more muscle mass have a more favourable metabolic phenotype including better glucose regulation, cholesterol levels, and blood pressure.^48,49^ Additionally, increased muscle mass has been shown to have a protective effect on vascular structure and function.^50,51^ Finding an inverse association between FFM and CHD events is important as it indicates that rather than solely recommending weight loss to decrease CVD risk, it is just as important to emphasize the importance of methods to alter body composition/improve muscle mass such as isometric exercise and weight training. A recommendation for exercise to improve muscle mass, rather than a focus solely on weight loss, may be a more clinically useful and attainable goal for prevention of heart disease in patients at risk.^22,52^

Waist-to-height ratio and waist circumference were included in the analysis as alternative measures of adiposity because they reflect central adiposity (to a certain degree) whereas FM reflects overall adiposity. Both waist-to-height ratio and waist circumference have been shown to predict FM and visceral adipose tissue.^19,53^ In the current study, waist-to-height ratio and waist circumference were shown to be significantly associated with increased risk of hard CHD events. Waist-to-height ratio was a better predictor of hard CHD events when compared with waist circumference and was slightly better than FM and FFM. The model that included both estimated FFM and waist-to-height ratio was the best predictor for hard CHD events, as evidenced by the highest LR χ^2^; thus, the combination of FFM and waist-to-height ratio can be surmised to most accurately reflect lean mass and visceral adiposity. These findings are similar to prior studies, which also showed a significant relationship between waist-to-height ratio and CVD risk.^54-56^ In individuals with hypertension and without pre-existing CVD, waist-to-height ratio predicted CVD incidence better than waist circumference and BMI.^56^ Additionally, in individuals with Type 2 diabetes, waist-to-height ratio had the strongest association with CVD events when compared with waist-to-hip ratio, waist circumference, and BMI.^55^ Waist-to-height ratio was also a stronger predictor of heart failure hospitalization when compared with BMI.^57^

Given the new recommendation to use waist-to-height ratio for diagnosing obesity^21^ and the European Society of Cardiology’s endorsement of assessing additional body composition measures to evaluate cardiometabolic risk,^22^ these findings highlight the value of incorporating alternative anthropometric measurements, including the waist-to-height ratio in combination with estimated FFM, rather than BMI to assess and monitor CVD risk. While a waist-to-height ratio cutoff of >0.5 has been proposed for diagnosing obesity,^21^ there are no studies that specifically exam waist-to-height ratio cut-offs in older adults. A recent study in young adults determined that a higher cut-off of 0.58 was more appropriate for determining metabolic syndrome.^58^ Future studies should explore assessing waist-to-height ratio and FFM to determine appropriate cut-off points for their use in the clinical setting.

BMI was not associated with increased risk of hard CHD events in our regression model. The literature regarding BMI and CVD is mixed, with some studies finding a decreased risk of CVD among individuals with higher BMIs, while others find a positive relationship between BMI and CVD incidence.^59,60^ Research examining CVD risk and BMI suggests the association is largely mediated by other risk factors such as low cardiorespiratory fitness, dyslipidaemia, hypertension, and diabetes mellitus, with a heterogeneity of risk across BMI values.^9,10,61,62^ These findings represent the limitations of BMI as a measure of adiposity and the limitations of BMI in characterizing adipose tissue quality and distribution.^59,60^ Including an analysis of BMI in the present study is important as studies comparing both BMI and other anthropometric measures of adiposity, and body composition measures, are scarce, while this study assesses body composition variables such of FM, FFM, and anthropometric measures of waist-to-height ratio, waist circumference, and BMI as risk factors for developing CHD events in a single population.^59^

There are many strengths of the present study as it includes a large, well-characterized multi-ethnic sample without clinically apparent CVD at baseline which lends to the generalizability of findings to a healthy population. Further this study developed a validated equation to measure FFM and FM tested across various ethnic groups. The inclusion of BMI in the analyses allows for comparison between two distinct measurements of obesity. Further, the equations derived from estimating FFM and FM at Exam 5 from the BIA values are excellent predictors of these quantities in NHANES 2003 and MESA Exam 1 cohorts. Limitations of the study include the inability to make causal inferences between the body composition measurements and hard CHD events from an observational study, limiting the outcome to hard CHD events, and given that the MESA participants are restricted in age range from 45 to 84 years findings may not be generalizable to younger people. Another limitation is that body fat distribution and adipose tissue, both of which play a critical role in determining cardiometabolic risk, was not measured.^4,11-18^ However, waist-to-height ratio and WC were included in this study as surrogates for body fat distribution.^53^

## Conclusions

Higher estimated FM, waist-to-height ratio, and waist circumference were associated with increased risk for hard CHD events, while higher estimated FFM was associated with decreased risk for hard CHD events. Future research is needed to investigate effective strategies to improve the implementation of lifestyle interventions to both build muscle and minimize adiposity in preventing CVD. The findings in this study also strengthen the call for the inclusion of alternative methodologies to define obesity and measure body composition in the clinical setting.

## Supplementary Material

supplementary

Supplementary material is available at *European Journal of Preventive Cardiology*.

## Figures and Tables

**Table 1:** Estimated DEXA and MESA Exam 1 fat-free mass compared with DEXA NHANES 2003 fat-free mass

	Males		Females	
	Mean	SD	Mean	SD
Fat-free mass				
Estimated MESA (*n* = 3210)	60.78	8.73	43.05	6.60
Estimated NHANES (*n* = 1284)	60.62	9.42	42.88	7.17
DEXA NHANES (*n* = 1284)	59.38	10.03	42.65	8.00

The estimated MESA Exam 1 FFM and estimated NHANES FFM are computed using the derived estimating equation from the MESA Exam 5.

DEXA, dual-energy x-ray absorptiometry; MESA, multi-ethnic study of atherosclerosis; NHANES, National Health and Nutrition Examination Survey; FFM, fat-free mass.

**Table 2:** Baseline characteristics of MESA Exam 1 participants (*N* = 6785)

	Sex	
	Female	Male
Total number of participants	3585 (52.8%)	3200 (47.2%)
Fat mass (kg)	30.66 (11.56)	23.35 (8.82)
Fat-free mass (kg)	43.05 (6.59)	60.78 (8.72)
Weight (kg)	73.71 (17.19)	84.13 (15.75)
Height (m)	1.60 (0.07)	1.74 (0.08)
Body mass index (kg/m^2^)	28.76 (6.23)	27.85 (4.45)
Waist (cm)	97.14 (16.03)	99.26 (12.24)
Waist-to-height ratio	0.61 (0.10)	0.57 (0.07)
Age at Exam 1	62.12 (10.27)	62.18 (10.22)
Race/ethnicity		
White	1360 (37.9%)	1254 (39.2%)
Chinese American	412 (11.5%)	389 (12.2%)
African American	1042 (29.1%)	838 (26.2%)
Hispanic	771 (21.5%)	719 (22.5%)
Taking diabetes medication		
No	3263 (91.0%)	2865 (89.5%)
Yes	322 (9.0%)	335 (10.5%)
Cigarettes: smoked in last 30 days		
No	3165 (88.3%)	2733 (85.4%)
Yes	420 (11.7%)	467 (14.6%)
HDL cholesterol (mg/dL)	56.26 (15.29)	45.07 (11.78)
LDL cholesterol (mg/dL)	199.59 (35.62)	188.05 (34.83)
Any lipid-lowering medication		
No	2997 (83.8%)	2672 (83.7%)
Yes	580 (16.2%)	521 (16.3%)
Systolic blood pressure (mmHg)	127.07 (23.21)	126.05 (19.36)
Any hypertension medication		
No	2203 (61.5%)	2052 (64.1%)
Yes	1379 (38.5%)	1148 (35.9%)
Family history of heart attack		
No	2028 (56.6%)	2033 (63.5%)
Yes	1557 (43.4%)	1167 (36.5%)

Family history includes parents, children, or siblings.

HDL, high-density lipoprotein; LDL, low-density lipoprotein; MESA, multi-ethnic study of atherosclerosis.

**Table 3:** Measures of body composition as predictors of hard coronary heart disease events at 10 years (*N* = 6785)

Variable	Hazard ratio	95% confidence interval	*P*-value
Model 1 (LR *χ*^2^ = 345.77)			
Fat-free mass (11.71 kg)	0.24	0.08, 0.76	0.014
Fat mass (10.98 kg) Model 2 (LR *χ*^2^ = 349.92)	1.64	1.09, 2.48	0.019
Waist-to-height ratio (0.089) Model 3 (LR *χ*^2^ = 325.41)	1.21	1.08, 1.35	0.001
Waist circumference (14.41 cm) Model 4 (LR *χ*^2^ = 327.68)	1.14	1.03, 1.28	0.016
Body mass index (5.48 kg/m^2^) Model 5 (LR *χ*^2^ = 358.24)	1.08	0.97, 1.21	0.158
Fat-free mass	0.26	0.11, 0.65	0.004
Waist-to-height ratio Model 6 (LR *χ*^2^ = 358.55)	1.31	1.16, 1.49	*P* < 0.001
Fat-free mass	0.13	0.04, 0.38	*P* < 0.001
Waist circumference	1.38	1.20, 1.60	*P* < 0.001

Hard CHD: myocardial infarction, resuscitated cardiac arrest, and fatal CHD. Each model was adjusted for age, sex, race/ethnicity, diabetes history, smoking history, HDL cholesterol, LDL cholesterol, lipid-lowering medication use, systolic blood pressure, hypertension medication use, and family history of heart attack. Hazard ratios presented are for 1 SD unit change in each variable, which are presented in parentheses.

CHD, coronary heart disease; LR, likelihood; HDL, high-density lipoprotein; LDL, low-density lipoprotein; MESA, multi-ethnic study of atherosclerosis; LR *χ*^2^, likelihood ratio χ^2^.

## Data Availability

The data underlying this article will be shared on reasonable request to the corresponding author.
