# Supplementary material for: Examining the relationship between anthropometric and body composition measures with coronary heart disease events: the multi-ethnic study of atherosclerosis
Source: Eur J Prev Cardiol. Author manuscript; Available in PMC 2026 Jul 13. (PMC13360764; doi:10.1093/eurjpc/zwaf212)
Supplement: supplementary [file NIHMS2182653-supplement-supplementary.docx]

| Supplementary Table 1: Comparison of MESA exam 1 participants (N=3210) to the NHANES 2003 participants (N=1284) | | | | |
| --- | --- | --- | --- | --- |
|  | Males | | Females | |
|  | Mean | SD | Mean | SD |
| Age |  |  |  |  |
| MESA | 62.19 | 10.21 | 62.14 | 10.25 |
| NHANES | 65.13 | 12.10 | 65.48 | 12.46 |
| Weight (kg) |  |  |  |  |
| MESA | 84.13 | 15.75 | 73.71 | 17.20 |
| NHANES | 85.45 | 17.89 | 74.46 | 18.46 |
| Height (meters) |  |  |  |  |
| MESA | 1.74 | 0.08 | 1.60 | 0.07 |
| NHANES | 1.74 | 0.08 | 1.60 | 0.07 |

| Supplementary Table 2: Measures of body composition as Predictors of Hard CHD Events at 10 Years (N = 6,785) | | | |
| --- | --- | --- | --- |
| Variable | Hazard Ratio | 95% confidence interval | P-value |
| Model 1 (LR Chi2 = 345.77) |  |  |  |
| Fat Free Mass (10 kg) | 0.76 | 0.61, 0.95 | 0.014 |
| Fat Mass (10 kg) | 1.20 | 1.03, 1.40 | 0.019 |
| Model 2 (LR Chi2 = 349.92) |  |  |  |
| Waist to Height Ratio | 8.32 | 2.39, 28.98 | 0.001 |
| Model 3 (LR Chi2 = 325.41) |  |  |  |
| Waist Circumference | 1.01 | 1.00, 1.02 | 0.016 |
| Model 4 (LR Chi2 = 327.68) |  |  |  |
| Body Mass Index | 1.02 | 0.99, 1.04 | 0.158 |
| Model 5 (LR Chi2 = 358.24) |  |  |  |
| Fat Free Mass (10 kg) | 0.77 | 0.65, 0.92 | 0.004 |
| Waist to Height Ratio | 21.18 | 5.205, 85.39 | P<0.001 |
| Model 6 (LR Chi2 = 358.55) |  |  |  |
| Fat Free Mass (10 kg) | 0.67 | 0.54, 0.83 | P<0.001 |
| Waist Circumference (cm) | 1.02 | 1.01, 1.03 | P<0.001 |
| Hard CHD: myocardial infarction, resuscitated cardiac arrest, and fatal CHD. Each model was adjusted for age, sex, race/ethnicity, diabetes history, smoking history, high-density lipoprotein cholesterol, low-density lipoprotein cholesterol, lipid lowering medication use, systolic blood pressure, hypertension medication use, and family history of heart attack. LR Chi2: Likelihood Ratio Chi-Square | | | |

| Supplementary Table 3: Anthropometric measures of body composition categorized into quartiles as predictors of hard CHD events at 10 Years (N = 6,785) | | | |
| --- | --- | --- | --- |
| Variable | Hazard Ratio | 95% confidence interval | P-value |
| Waist to Height Ratio |  |  |  |
| 2 | 1.20 | 0.88, 1.62 | 0.245 |
| 3 | 1.41 | 1.05, 1.91 | 0.024 |
| 4 | 1.54 | 1.12, 2.11 | 0.008 |
| Waist Circumference |  |  |  |
| 2 | 1.21 | 0.89, 1.65 | 0.225 |
| 3 | 1.22 | 0.89, 1.67 | 0.207 |
| 4 | 1.34 | 0.97, 1.85 | 0.075 |
| Body Mass Index |  |  |  |
| 2 | 1.14 | 0.85, 1.51 | 0.378 |
| 3 | 1.12 | 0.83, 1.50 | 0.450 |
| 4 | 1.28 | 0.95, 1.75 | 0.105 |
| Hard CHD: myocardial infarction, resuscitated cardiac arrest, and fatal CHD. Each model was adjusted for age, sex, race/ethnicity, diabetes history, smoking history, high-density lipoprotein cholesterol, low-density lipoprotein cholesterol, lipid lowering medication use, systolic blood pressure, hypertension medication use, and family history of heart attack. LR Chi2: First quartile was used as the reference group. | | | |
